# Talonavicular-cuneiform arthrodesis for the treatment of Müller-Weiss: mid-term results of 15 cases after 5 years

**DOI:** 10.1186/s12891-023-06293-1

**Published:** 2023-03-10

**Authors:** Wenbo Bai, Yinghao Li, Guodong Shen, Hongning Zhang, Xue Li, Yongzhan Zhu

**Affiliations:** Department of Foot and Ankle Orthopedics, Foshan Hospital of Traditional Chinese Medicine Affiliated to Guangzhou University of Chinese Medicine, Foshan, 528000 Guangdong China

**Keywords:** Müller-Weiss, Talonavicular-cuneiform arthrodesis, Mid-term results, Operative treatment

## Abstract

**Background:**

There is no gold standard for the operative treatment of patients with Müller-Weiss disease (MWD). This study reports the mid-term follow-up results for at least 5 years following talonavicular-cuneiform (TNC) arthrodesis for Müller-Weiss disease.

**Methods:**

A total of 15 patients undergoing TNC arthrodesis for MWD were retrospectively reviewed between January 2015 and August 2017. Two senior doctors assessed the radiographic results twice at each visit (preoperative, three months after the operation, and final follow-up). The clinical results and complications from preoperative and final follow-up were recorded.

**Results:**

The mean follow up period was 74.0 (range 64 to 90) months. The calcaneal pitch angle, lateral Meary's angle, anteroposterior (AP) Meary's angle, AP talocalcaneal angle, and talonavicular coverage were significantly different before and three months after the operation (*p* < 0.05). There was no significant difference between the radiographic results of three months after the operation and the final follow-up (*p* > 0.05). The radiological measurements of the two senior doctors were calculated and found to be moderate to strong (ICC:0.899–0.995). The AOFAS, VAS, and SF-12 scores significantly improved at the last follow-up compared to those before the operation (*p* < 0.05). Two patients experienced early complications, four had late complications, and one underwent a second operation of midfoot fusion with calcaneal osteotomy.

**Conclusion:**

This research confirms that using TNC arthrodesis for the treatment of MWD can substantially improve the clinical and radiographic results. These results were maintained until mid-term follow-up.

## Introduction

Müller-Weiss disease (MWD) is a rare condition in clinical practice. Clinically, MWD affects the navicular, a wedge-shaped bone that articulates with five tarsal bones to form syndesmotic joints. And the navicular is also a potential site for spontaneous osteonecrosis in adults [1]. There is substantial controversy regarding the etiology and pathogenesis of MWD, including primary osteonecrosis [2], sequelae of undiagnosed navicular stress fractures [3], necrosis of traumatic or biomechanical origin [4], and abnormal evolution of Kohler’s disease [5]. Delayed ossification of the tarsal navicular and abnormal force distribution pattern proposed by Maceira et al. [6] is the most accepted cause of MWD. The biomechanical change consists of several features, including lateral navicular compression, medial longitudinal arch collapse, lateral displacement of the talar head, hindfoot varus deformity, and the gradual development of arthritis in the peri-navicular joints, producing a paradoxical pes planus varus [1, 6–8]. Radiographic imaging of MWD demonstrates a comma-shaped or hourglass-shaped navicular on the AP view and narrowing and sclerosis on the lateral view [8, 9].

Due to the complexity of etiological factors and mechanical mechanisms of MWD, there is no gold standard treatment technology for patients with MWD. Almost all studies on MWD treatment support initial conservative treatments, including stretching exercises, magnetotherapy, and functional orthoses [3, 8]. In addition, surgical interventions, including internal fixation of navicular, percutaneous decompression, calcaneal osteotomy, isolated talonavicular (TN) arthrodesis, talonavicular-cuneiform (TNC) arthrodesis or triple arthrodesis are considered if symptoms persist for more than six months. However, there has yet to be a consensus about the best option for achieving satisfactory results and minimizing complications. Scholars are yet to determine whether arthrodesis should be extended to the complex naviculocuneiform, calcaneocuboid, or subtalar joints [1, 10–16]. Maceira et al. initially suggested the use of TNC arthrodesis, and subsequent studies have developed and described different techniques for TNC arthrodesis to the restoration of the medial arch [1, 12, 16–20]. But its safety and efficacy are still questioned, and the follow-up time is too short, the complications, final fixation results, clinical efficacy and bone resorption of bone grafts may not be observed in TNC arthrodesis.

Therefore, this study aimed to explore whether TNC arthrodesis could effectively improve the clinical efficacy of MWD based on a mid-term follow-up of up to five years. This study used radiographic data to determine whether TNC arthrodesis could improve foot alignment and whether the results could be maintained until mid-term follow-up. Additional objectives were to observe and analyze early or late complications after TNC arthrodesis. To the best of our current knowledge, it is the first study reporting mid-term follow-up results for TNC arthrodesis of MWD.

## Methods

### Patients

Patients were included in this study if: (1) diagnosis was based on clinical evaluation and radiologic findings on weight-bearing AP and lateral views; (2) had at least 5 years postoperative follow-up; (3) the conservative treatment was ineffective for more than 6 months; (4) the surgical method is TNC arthrodesis; and (5) they had only unilateral limb surgery. Patients were excluded from the study if: (1) had a history of Kohler disease; (2) a previous traumatic or stress fracture of the navicular; (3) had rheumatoid arthritis; and (4) incomplete radiographic data or missing follow-up data.

**Fig. 1:**
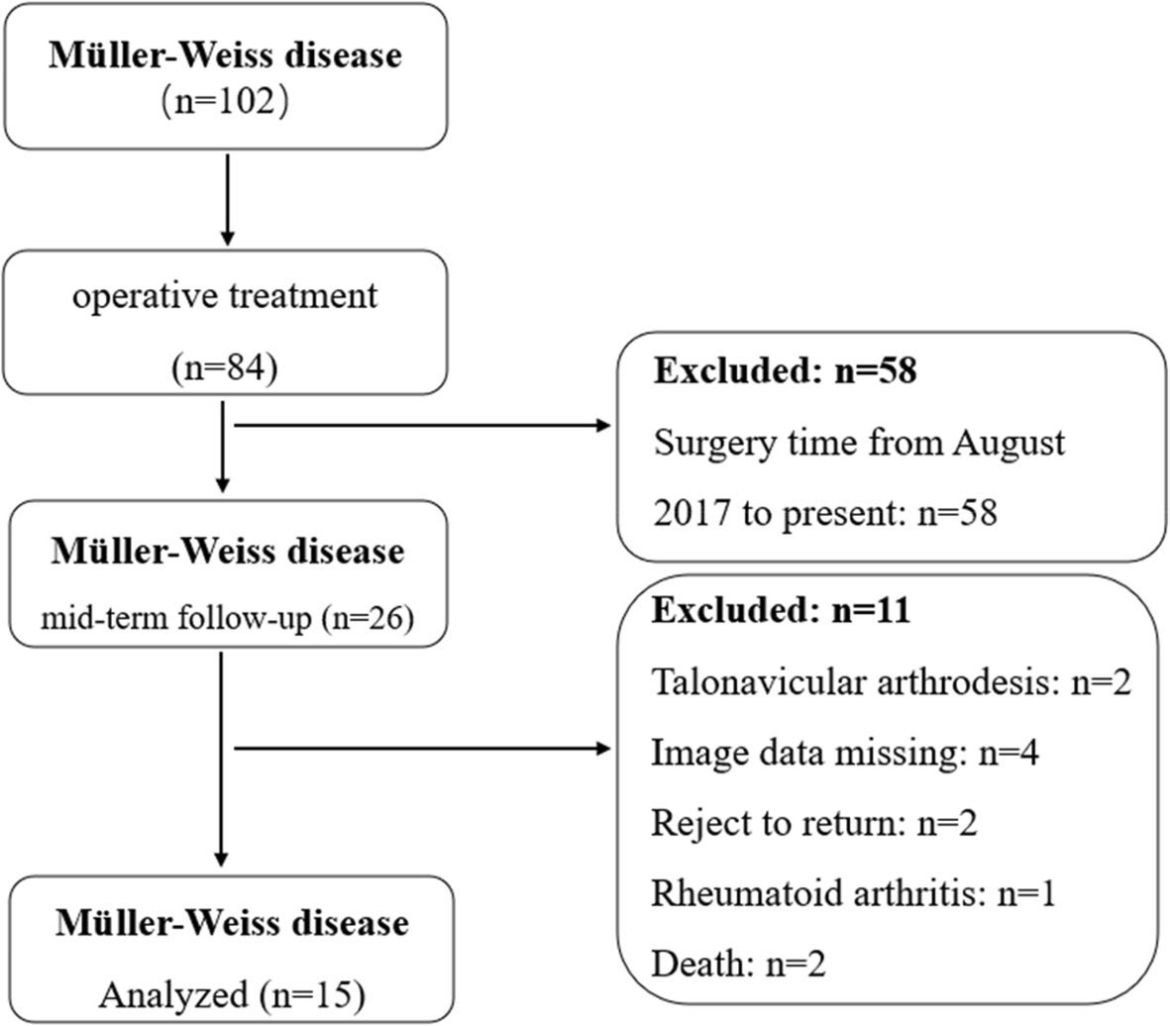
This figure shows the number of patients included in this study

In total, 26 patients underwent operative treatment for the dysfunction and pain caused by MWD between January 2015 and August 2017. This study reviewed 15 patients who had undergone TNC arthrodesis at the mid-term follow-up and met the inclusion criteria. The patient enrollment is described in Fig. 1. This study was approved by the institutional review board. The participants used in this study provided informed consent. Two senior doctors performed all operative procedures in line with standardized protocols.

### Operative procedure

As determined by the individual situation, an autogenous tricortical bone graft of appropriate size was harvested from the ipsilateral iliac crest. Cancellous bone was harvested with the smallest osteotome possible. A longitudinal dorsal incision was made lateral to the extensor hallucis longus tendon with an interface between the extensor hallucis longus tendon and the dorsalis pedis artery, both of which were retracted correspondingly. The soft tissue was distracted by a lamina spreader to expose the talonavicular and navicular-cuneiform joints. The talonavicular and navicular-cuneiform joints were distracted using a Hintermann distractor over separate K-wires. The articular surfaces were debrided in situ with cartilage shovels to the subchondral bone. A K-wire was used to drill the subchondral sclerotic bone plate into a favaginous condition for fusion. Then bite off the excess osteophyte from the lateral 4-corners. The plantar ligament and plantar soft tissue of the navicular are loosened with a sharp knife, leaving only the insertion point of the posterior tibial tendon. The whole debridement process created a relative space around the navicular bone. Subsequently, a periosteal detacher was pressed against the lateral bony protrusion of the navicular bone to rotate the bone outwards to the original top location. Once the reduction was deemed satisfactory by direct visualization, two to three crossing K-wires were used for temporary fixation. After the demonstration of the corrected medial longitudinal arch on the C-arm, the lateral half of the navicular bone (including the talonavicular and navicular-cuneiform joints involved in the necrotic lesion) was excised using an osteotome to form a broad dorsal trapezoid laterally and a rectangular longitudinal bone bed. And the modified tricortical iliac bone block was inserted into the space between the talus and the cuneiforms. Finally, two hollow lag screws and a dorsal LCP were used to arthrodese the talonavicular-cuneiform joints. A transverse Herbert screw was used (where needed) to fix the bone block and medial navicular bone. The wound was closed after packing the previously acquired cancellous bone to smooth the defect gaps.

Postoperatively, a protective non-weight bearing short-leg plaster cast was applied for 6 weeks, after which weight-bearing was gradually allowed as tolerated.

### Clinical and radiographic assessment

Clinical assessments and analyses were performed on the patients. The American Orthopedic Foot and Ankle Society (AOFAS), Visual Analogue score (VAS), and SF-12 [21] were used to evaluate the function before the operation and at the last follow-up. The mental component score (MCS) and physical component score (PCS) were calculated using the SF-12 based on the Ware et al. Manual [22]. The final follow-up visits were performed from August to December 2022.

**Fig. 2:**
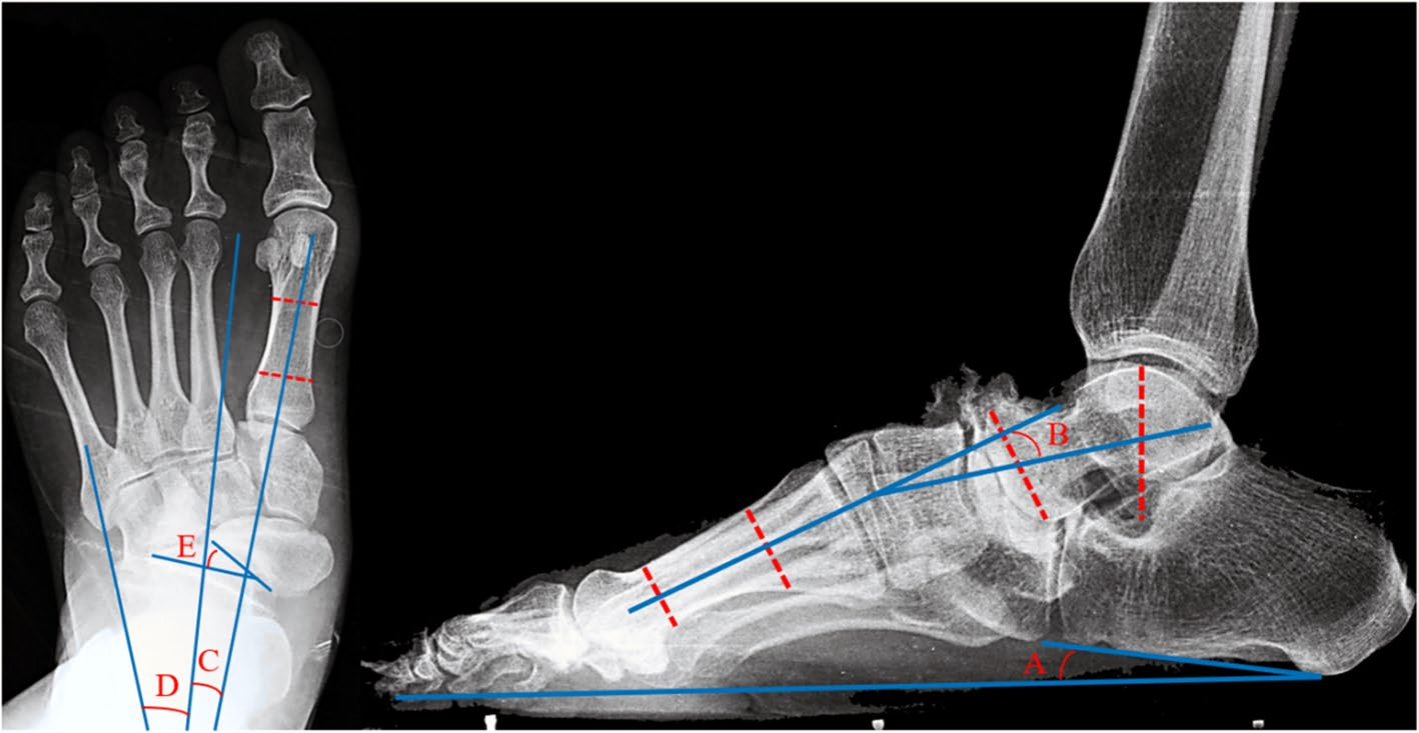
Measurement parameters on weight-bearing AP and lateral views. **A** The calcaneal pitch angle. **B** The lateral Meary's angle (positive sign = dorsal intersection; negative sign = plantar intersection). **C** Ap Meary's angle (positive sign = first metatarsal abduction; negative sign = adduction). **D** Ap talocalcaneal angle. **E** The talonavicular coverage (positive sign = navicular bone in valgus; negative sign = navicular bone in varus)

The foot was examined radiologically in the weight-bearing AP and lateral view. Calcaneal pitch angle, lateral Meary's angle, AP Meary's angle, AP talocalcaneal angle, and talonavicular coverage were measured twice by two different senior doctors at each visit (preoperative, three months after the operation and final follow-up). A successful fusion was defined as a painless foot during weight-bearing and trabeculation across the fusion line on radiography. These parameters measured on the weight-bearing AP and lateral views of the foot are shown in Fig. 2.

### Statistical analysis

The statistical analyses were performed using SPSS 25.0. All continuous data were tested for normality using the Shapiro–Wilk test and presented as the mean ± standard deviation for normally distributed data and median (25^th^ percentile and 75^th^ percentile) for nonnormal distributed data. An intragroup comparison of data with normal distribution was performed using paired sample t-tests. An intragroup comparison of data with abnormal distribution was performed using the nonparametric Wilcoxon signed-rank test. The level of significance was set at 0.05 for all analyses. Interrater agreement was evaluated using the intraclass correlation coefficient (ICC).

## Results

### Demographic data

Degeneration was observed in 15 (100%) talonavicular joints, 13 (83.6%) navicular-cuneiform joints, 8 (53.3%) calcaneocuboid joints, and 3 (20%) subtalar joints. CT examination revealed degeneration in 12 (80.0%) “4-corners”, the junction between the anterior process of the calcaneus, the inferior border of the talar head, and the lateral corner of the navicular and cuboid. The mean follow-up time was 74.0 ± 8.43 (range, 64–90) months. The demographic descriptions of the patients are displayed in Table 1.

**Table 1:** Patient information

Case	Age (y)	Gender	BMI	Side	Onset time (y)	MWD stage	Follow-up (mo)	Early complications	Late complications
1	61	W	23.5	L	16	III	90	None	medial navicular pain
2	63	W	22.6	L	4	III	84	None	None
3	62	W	26.8	R	10	III	84	None	Lateral forefoot pain
4	48	W	30.2	L	3	IV	80	None	None
5	64	M	27.3	L	20	IV	78	None	None
6	53	W	24.6	L	13	IV	77	None	medial navicular pain
7	61	W	28.2	L	1	III	77	None	None
8	63	W	24.0	L	8	III	76	Medial dorsalis pedis numbness	None
9	45	M	27.5	L	7	III	72	None	Calf muscle atrophy
10	39	W	30.4	L	5	IV	68	None	None
11	60	W	28.7	L	5	III	66	None	None
12	41	W	23.6	R	2	IV	65	None	None
13	58	W	29.3	L	8	IV	65	Hardware stimulation	None
14	63	W	24.0	L	7	IV	64	None	None
15	70	W	21.6	R	3	III	64	None	None

### Clinical results

Unlike the preoperative scores, the AOFAS, VAS, SF12-PCS, and SF12-MCS scores improved at the last follow-up (*p* < 0.05). The mean AOFAS score increase was 48.67, and the median VAS score decrease was 6. On the other hand, the SF12-PCS and SF12-MCS increased by 24.33 and 19.87, respectively (Table 2).

**Table 2:** Preoperative and last follow-up clinical outcomes

Items	Preoperative	95% CI	Follow-up	95% CI	*P* value
VAS	7.00(6.00,8.00)	6.49—7.37	1.00(1.00,2.00)	0.81—2.25	0.001
AOFAS	41.53 ± 9.47	36.28—46.78	90.20 ± 5.97	86.88—93.51	0.000
SF12-PCS	26.53(24.26,34.58)	25.96—31.73	50.86(49.74,56.91)	50.84—54.59	0.001
SF12-MCS	35.61 ± 4.26	33.25—37.97	55.48 ± 3.6	53.47—57.48	0.000

### Radiologic results

**Fig. 3:**
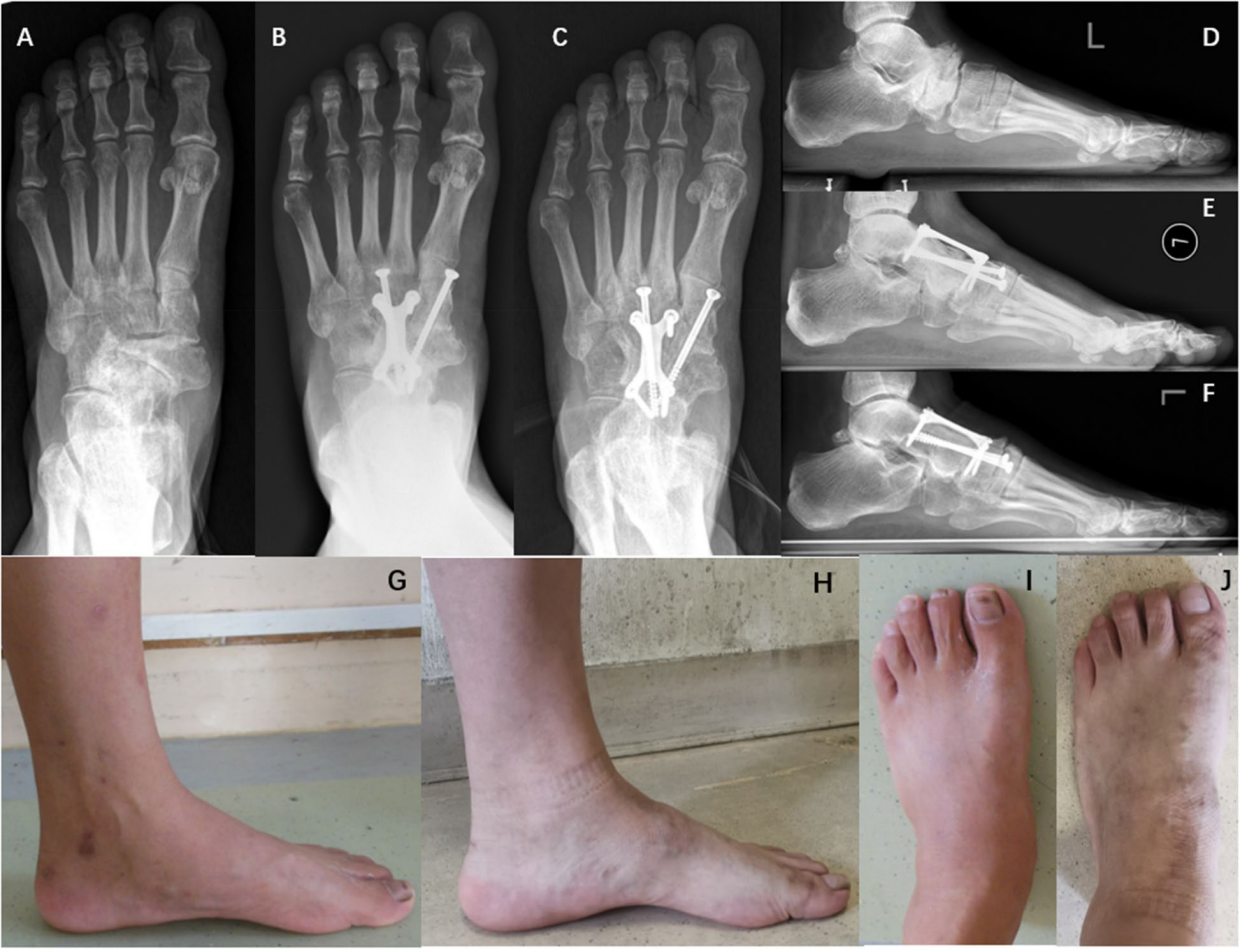
Patient No. 11. **A**-**C** Weight-bearing AP X-ray films before the operation, 3 months after the operation and last follow-up. Improved forefoot abduction, no nonunion and bone resorption; **D**-**F** Weight-bearing lateral X-ray films before the operation, 3 months after the operation and last follow-up. And lateral Meary's angle backs to normal; **G**-**J** Appearance before operation and last follow-up

Table 3 shows the radiological measurements taken by two different orthopedic doctors. The results of this study found a significant difference between follow-up before the operation and follow-up after three months in terms of calcaneal pitch angle, lateral Meary's angle, AP Meary's angle, AP talocalcaneal angle, and talonavicular coverage (*p* < 0.05). However, there were no significant differences between follow-up after three months and the final follow-up (*p* > 0.05, Table 3, Fig. 3).

**Table 3:** Results of radiologic measurements by two different orthopedic doctors

	Preoperative	95% CI	Postoperative (3 mo)	95% CI	*P* value	Last follow-up	95% CI	*P* value
Doctor A								
Calcaneal pitch angle	14.99 ± 7.10	11.05—18.93	21.22 ± 5.05	18.42—24.02	0.000	20.43 ± 5.35	17.46—23.39	0.173
Lateral Meary's angle	-4.00 ± 10.71	-9.94—1.92	1.47 ± 4.91	-1.24—4.19	0.032	1.89 ± 5.06	-0.91—4.69	0.237
Ap Meary's angle	9.19 ± 9.19	4.10—14.28	3.00(1.50,7.32)	2.28—6.73	0.015	2.40(1.12,9.67)	1.98—6.97	0.551
Ap talocalcaneal angle	18.75 ± 6.85	14.95—22.54	22.73 ± 4.57	20.20—25.27	0.013	23.31 ± 5.08	20.49—26.12	0.498
Talonavicular coverage	-14.83 ± 7.73	-19.12—-10.55	-3.00(-4.25, -1.30)	-4.19—-1.92	0.001	-3.00(-4.12, -1.00)	-3.89—-1.55	0.136
Doctor B								
Calcaneal pitch angle	14.94 ± 6.65	11.25—18.63	21.60 ± 5.26	18.69—24.52	0.000	20.90 ± 5.62	17.79—24.02	0.106
Lateral Meary's angle	-4.43 ± 11.14	-10.60—1.73	0.95 ± 4.45	-1.51—3.42	0.040	1.75 ± 4.61	-0.79—4.30	0.075
Ap Meary's angle	9.26 ± 9.59	3.94—14.57	3.00(0.48,9.12)	1.96—6.43	0.009	2.30(1.00,9.00)	1.95—6.70	0.778
Ap talocalcaneal angle	17.38 ± 6.85	13.58—21.17	23.40 ± 4.53	20.89—25.91	0.001	22.82 ± 5.03	20.03—25.61	0.463
Talonavicular coverage	-14.64 ± 7.33	-18.70—-10.58	-3.00(-4.00, -1.00)	-4.05—-1.59	0.001	-2.96(-4.00, -1.00)	-3.80—-1.52	0.182

*CI* Confidence Interval, *Postoperative (3 mo)* 3 months after the operation

And the radiological results among the two orthopedic doctors were calculated using the ICC, which was found to be moderate to strong (0.899–0.995) (Table 4).

**Table 4:** Radiologic results using ICC

	Preoperative(95% CI)	Postoperative(3 mo, 95% CI)	Last follow-up(95% CI)
Calcaneal pitch angle	0.991(0.973,0.997)	0.969(0.913,0.989)	0.965(0.901,0.988)
Lateral Meary's angle	0.995(0.986,0.998)	0.976(0.915,0.992)	0.991(0.975,0.997)
Ap Meary's angle	0.995(0.984,0.998)	0.956(0.878,0.985)	0.986(0.961,0.995)
Ap talocalcaneal angle	0.918(0.747,0.973)	0.926(0.794,0.974)	0.982(0.936,0.994)
Talonavicular coverage	0.964(0.897,0.988)	0.915(0.773,0.970)	0.899(0.726,0.965)

### Complications

No patient developed a deep infection, wound healing disorders, delayed bony union, nonunion, and adjacent joint degeneration during the complete follow-up. Two patients developed early postoperative complications (within 2 years), including medial dorsal pedis numbness (1/15, 6.6%) and hardware stimulation (1/15, 6.6%). One patient developed numbness at the incision at the 3-month follow-up. Suspected deep peroneal nerve injuries were treated with neurotrophic drugs and observed closely. The other patient experienced hardware stimulation during the 1-year follow-up. Considering the bony fusion of the talonavicular-cuneiform joint, the internal fixation of the patient was removed. At the last follow-up, patients recovered completely without any discomfort.

**Fig. 4:**
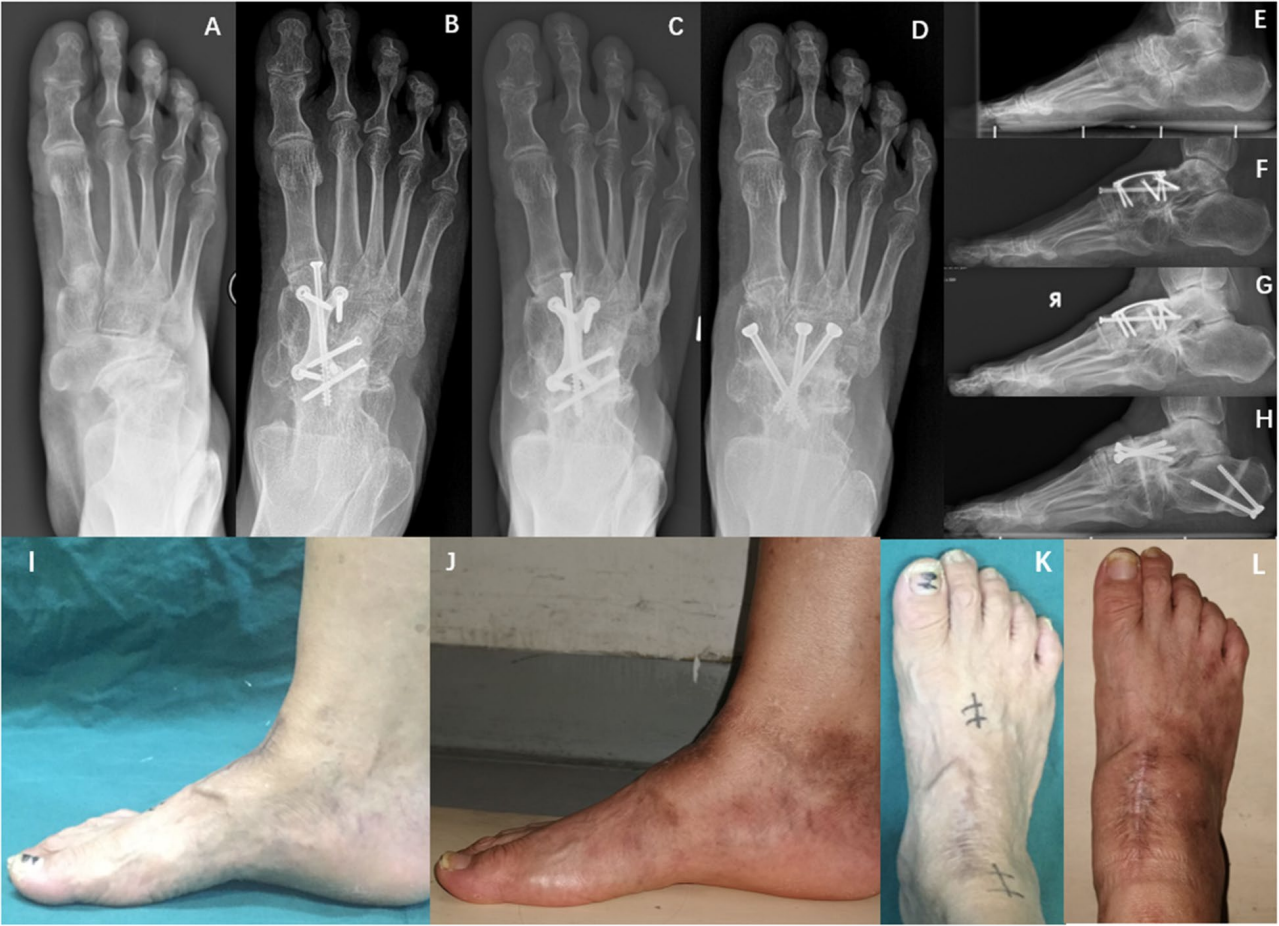
Patient No. 3. **A**-**C** Weight-bearing AP X-ray films before the operation, 3 months and 4 years after the operation. And mild forefoot supination deformity; **D** With midfoot osteotomy, final follow-up; **E**-**H** Weight-bearing lateral X-ray films before the operation, 3 months and 4 years after the operation, and last follow-up. And cooperating with calcaneal osteotomy to correct hindfoot varus; **I**-**L** Appearance of 4 years after operation and revision operation

Four patients developed late postoperative complications (2 years or more), including mild atrophy of the calf muscles (1/15, 6.6%), medial navicular pain (2 /15,13.3%), and lateral forefoot pain (1/15, 6.6%). One patient developed mild atrophy of the calf muscle on the affected side without clinical symptoms. As a result, a rehabilitation specialist was consulted to guide the patient with mild atrophy on specific rehabilitation training. Two patients had walking pain on the medial part of the navicular bone, which was relieved by rest. The VAS scores were all 3 points. But the patients were satisfied with our treatment effect. This study recommended that patients wear comfortable sports cotton-soled shoes to return to normal life and exercise. One patient developed lateral pain in the forefoot 4 years after the operation. The appearance of the affected foot showed slight varus of the hindfoot and supination of the forefoot. And we had revision surgery on it with midfoot fusion and calcaneal osteotomy (Fig. 4).

## Discussion

Müller-Weiss disease is a complex and rare condition involving a sequence of foot deformities. We evaluated the degeneration of the calcaneocuboid (8/15,53.3%) and subtalar (3/15,20%) joints through preoperative CT, which mostly manifests as narrow joint space and transparent shadow of marginal bone, and its sensitivity was stronger than that of X-rays. However, in the clinical examination, there was no obvious tenderness and walking pain in the calcaneocuboid and subtalar joints, and the patient's main complaint was also chronic pain in the dorsomedial midfoot during weight bearing. In addition, Harnroongroj et al. [23] compared instances when conservative treatment methods of MWD succeeded and failed. Harnroongroj et al. determined that the degree of midfoot abduction represented by the AP Meary's angle and radiographic talonavicular arthritis promoted the failure of conservative treatment in MWD. Therefore, the proposed operative treatments aim to restore medial column height, correct forefoot abduction deformity, and arthrodesis of the involved bony structure to achieve a plantigrade, well-aligned foot.

This study comprised 8 patients with MWD stage III and 7 patients with MWD stage IV with varying degrees of rupture and compression of the navicular, showing peri-navicular osteoarthritis. It is believed that enhancing navicular stability using fusion can prevent the development of deformity, thereby relieving pain and restoring foot alignment. Triple arthrodesis can effectively reduce the pain caused by hindfoot and midfoot arthritis and deformity. Triple arthrodesis is mostly used in patients with MWD stage IV to V [15, 16]. However, we believe that triple arthrodesis may not be suitable for our patients. Fusion on subtalar and calcaneocuboid joints is unnecessary and is more likely to cause hindfoot stiffness and decreased foot function. Correspondingly, Maceira et al. [6] did not support using isolated TN arthrodesis because it could not resolve the joint incongruity in the naviculocuneiform joints, consolidation of TN arthrodesis is poor, and carriesa high risk of pseudoarthosis [17]. Unlike other joints in the foot, the naviculocuneiform and talonavicular joints bear the greatest stress and load transmission [24, 25]. Therefore, we agree with Maceira [1, 6, 10] that a strong fusion of the TNC may be a better option when the navicular ruptures, developing peri-navicular osteoarthritis.

In addition, a review of the English literature on the use of TNC arthrodesis for MWD was conducted. Table 5 shows that most scholars used TNC arthrodesis for stage III ~ IV MWD, and their short-term results are effective. This study agrees with Yu [19] on restoring the length of the medial column. However, reducing the medial half of the navicular is more important because the internal rotation of the medial fragment of the navicular is the main factor for the relative shortening of the medial column of the foot. Therefore, this study focused on restoring the navicular bone to the top of the medial longitudinal arch by rotating the bone outward. Reducing the medial half of the navicular should be a precondition for subsequent reconstruction of the lateral half of the navicular. Besides, Doyle et al. [26] found nine patients (9/12,75.0%) whose CT was showing degeneration at the "4 corners" and were staged at stage III and above. The findings of this study found that all patients with stage IV developed the "4 corners". It was assumed that as the flattening of the plantolateral aspect of the navicular, the talar head would then move laterally and inferiorly and impinges the calcaneal anterior process, further increasing midfoot lateral pressure, resulting in 4-corners degenerative changes, and even pes planus varus and cuboid sign. Therefore, we also emphasized the debridement of the “4 corners”, which is beneficial to the reduction of the talonavicular joint on the sagittal plane by cleaning the osteophyte on the anterolateral side of the talus head. And loosen the cervical ligament of anterior aspect of the tarsal sinus and clean the osteophyte around the calcaneocuboid joint, which is beneficial to the adduction and internal rotation reduction of the talus head.

**Table 5:** TNC arthrodesis for MWD in the English literature

Authors	feet	Stage	Follow-up (mo)	Results	Treatment concept
Fernandez et al. (2004) [1]	–	–	–	Alleviating the pain and excellent results	First advocacy of TNC arthrodesis for MWD
Yu et al. (2012) [19]	7	III 4	Median 22.0(range 3 to 38)	AOFAS score 82 (range 68 to 97); screw breakage (1 case)	Restoring the length of the medial column
		IV 1			
		V 2			
Cao et al. (2012) [17]	9	III 9	Mean 22.4 (range 12 to 52)	AOFAS score 90.9 ± 2.1; pain-free	“V” shaped osteotomy
Cao et al. (2017) [18]	14	III 14	Mean 51.7 (range 12 to 90)	AOFAS score 90.1 ± 2.0; pain-free	“V” shape osteotomy and autoallergic iliac bone graft
Zhang et al. (2017) [16]	6	IV 6	Mean 8.20 (range 2 to 28)	AOFAS score 86.2 ± 3.49; pain-free	Tricortical autogenous graft to fusion

The mid-term results of this study were satisfactory, with a mean AOFAS score of 90.20 ± 5.97 (95% CI, 86.88–93.51). Other studies with different treatment modalities reported postoperative AOFAS scores ranging from 82.0 to 90.9, as shown in Table 5. The SF12-PCS and MCS scores were calculated using standard U.S. scoring algorithms and compared to the general population [22]. These scores showed that the participants used in this study had favorable results (t = 3.118, *P* = 0.004; t = 5.861, *p* = 0.000), suggesting that participants recovered to ideal physical and mental health. All patients in this study recovered and returned to their daily activities.

In the present study, we also found that the incidence rate of calcaneocuboid degeneration and subtalar degeneration before the operation were 53.3 and 20.0%, respectively. However, during the last follow-up, X-ray degeneration was comparable to that before the operation, and the patients showed no pain symptoms. Subtalar varus is defined as lateralization of the head of the talus [6]. And this subtalar varus causes abnormal stress distribution, resulting in subtalar osteoarthritis. We believe that once the talus head is realigned to the normal position, the progression of subtalar osteoarthritis will be terminated. Internal rotation of the medial half of the navicular shortens the medial column of the foot, increasing plantar pressure at the lateral midfoot [6, 27]. The force from the forefoot is then focused on the calcaneocuboid joint, which will generate osteoarthritis. Postoperative reconstruction of the medial column will loosen the calcaneocuboid joint, preventing osteoarthritis. The talonavicular and naviculocuneiform joints are involved in the pathological process, whereas the subtalar and calcaneocuboid joints are secondary lesions. This study believes that TNC arthrodesis may delay or terminate the progression of calcaneocuboid or subtalar arthritis.

The radiographic findings showed a tendency toward normalization of foot physiology and anatomy following operative treatment, making it the most important finding of this study. On the one hand, scores collected three months after the operation were better than those measured preoperative (*p* < 0.05). As a result, this study improved medial column height and forefoot abduction deformity and reconstructed the alignment, meeting its proposed operative treatment goals. On the other hand, we didn’t observe a developed deformity at mid-term follow-up, nor do we note possible bone resorption, sclerosis, and nonunion with autologous bone grafts. And our early radiological results remained consistent after a mean follow-up of 74 months (Figs. 3 and 4). This finding suggested that the fixation method of “H”-shaped LCP combined with lag screws provides strong support for reconstructing foot alignment. In addition, the results confirm that the iliac bone graft is undoubtedly a safe and effective way of bone graft.

In the present study, adjacent joint degeneration did not develop after TNC arthrodesis. And in the outpatient follow-up, the pain symptoms of most patients became more and more painless with the passage of time. One patient developed lateral forefoot pain, characterized by walking pain at the base of the fifth metatarsal and the formation of the lateral corpus callosum of the forefoot 4 years after the operation (Fig. 4). Lui et al. [15] reported a similar complication. A pressure study showed increased lateral sole pressure and they suggested isolated calcaneal osteotomy to address the hindfoot varus deformity. And we believe that this complication could have been caused by insufficient attention to hindfoot varus preoperative and insufficient forefoot pronation during the operation resulting in shortening of the medial column. We agreed with Lui [15], but we also corrected the forefoot supination with an additional midfoot osteotomy. And the patient's AOFAS score reached 97 at the last follow-up, which was pain-free. In Maceira’s theory, hindfoot varus is a prerequisite for MWD diagnosis [6]. He also mentioned that hindfoot malalignment should be corrected when varus deformity is observed, with subtalar correction or calcaneal osteotomy [1, 6, 8]. Li and Myerson [10] performed a single calcaneal osteotomy incorporating a wedge and lateral translation in 14 feet with stage II to V MWD, and their results were also satisfactory. Therefore, we suggest that the sagittal plane alignment, coronal plane alignment, and foot rotation deformity should be completely corrected during the operation. At the same time, if severe hindfoot varus deformity is observed, calcaneal osteotomy can be used as an auxiliary operative method.

This study has several limitations. On the one hand, the patient sample size was small and had no controls. Therefore, there is a need to conduct multi-center clinical trials in the future. On the other hand, this study used clinical examinations and X-rays to assess the degree of postoperative bone fusion and arthritis without spiral CT scans. Future studies should use CT scans for asymptomatic follow-up patients.

In conclusion, this study showed a significant improvement in clinical and radiographic results after the treatment of MWD with TNC arthrodesis. These results were maintained until mid-term follow-up. Therefore, for patients with MWD who have peri-navicular osteoarthritis, even though it may be accompanied by mild subtalar or calcaneocuboid arthritis, TNC arthrodesis may be a good option.

## Data Availability

The datasets generated and analysed during the current study are available from the corresponding author on reasonable request.
